# Classical monocytes-low expressing HLA-DR is associated with higher mortality rate in SARS-CoV-2+ young patients with severe pneumonia

**DOI:** 10.1016/j.heliyon.2024.e24099

**Published:** 2024-01-09

**Authors:** Juan Sebastián Henao-Agudelo, Sebastian Ayala, Marisol Badiel, Andrés F. Zea-Vera, Lorena Matta Cortes

**Affiliations:** a Faculty of Health Sciences, Unidad Central del Valle del Cauca, Tuluá, Colombia; b Department of Internal Medicine, Universidad del Valle, Cali, Colombia; c Hospital Universitario del Valle Evaristo García, Cali, Colombia; d School of Basic Sciences, Universidad del Valle, Cali, Colombia; e LCIM//Division of Intramural Research, NIAID, National Institutes of Health, Bethesda, MD, USA

**Keywords:** HLA-DR, COVID-19, SARS-CoV-2, Monocytes, PD-L1, Pneumonia, Young adults

## Abstract

**Aims:**

This study aimed to investigate whether monocyte dysregulation and hyperinflammation serve as predictive markers for mortality in young patients with SARS-CoV-2 severe pneumonia.

**Methods:**

A prospective cohort study was conducted in a tertiary-level public University Hospital in Colombia. Forty young adults (18–50 years of age) with severe pneumonia and SARS-CoV-2 infection confirmed by qPCR, were enrroled. Serum cytokines and the monocyte phenotype profile, including PDL1/HLA-DR expression, were determined during the first 24 h of hospitalization. Routine laboratory parameters were measured throughout patient follow-up until either death or hospital discharge. We also included a cohort of twenty-five healthy control subjects.

**Key findings:**

Elevated levels of IL-10, IL-8, and IL-6 cytokines emerged as robust predictors of mortality in young adults with severe pneumonia due to SARS-CoV-2 infected. A descriptive analysis revealed a cumulative mortality rate of 30 % in unvaccinated and ICU-admitted patients. Patients who died had significantly lower expression of HLA-DR on their classical monocytes subsets (CD14^+^CD16^−^) than survivors and healthy controls. Lower expression of HLA-DR was associated with greater clinical severity (APACHE≥12) and bacterial coinfection (relative risk 2.5 95%CI [1.18–5.74]). Notably, the expression of HLA-DR in 27.5 % of CD14^+^/CD16^-^ monocytes was associated with a significantly lower probability of survival.

**Significance:**

The early reduction in HLA-DR expression within classical monocytes emerged as an independent predictor of mortality, irrespective of comorbidities. Together with PD-L1 expression and cytokine alterations, these findings support the notion that monocyte immunosuppression plays a fundamental role in the pathogenesis and mortality of young patients infected with SARS-CoV-2. These findings hold significant implications for risk assessment and therapeutic strategies in managing critically ill young adults with SARS-CoV-2 infection.

## Graphical abstract

**Figure:**
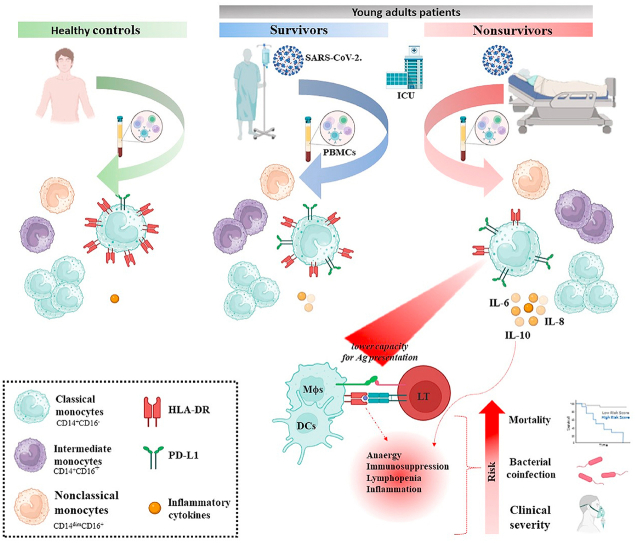

## 1 Introduction

In 2019, a new virus was responsible for the appearance of cases of severe pneumonia, named severe acute respiratory distress syndrome coronavirus type 2 (SARS-CoV-2), in the province of Wuhan, China [1]. After 3 years of the coronavirus disease 2019 (COVID-19) pandemic declaration, which killed more than 7 million worldwide, SARS-CoV-2 viruses was shown to infect all age groups. However, older adults (>65 years, according to the NIH designation), had the highest burden of morbidity and mortality globally [2]. Interestingly, young adults (between 18 and 50 years old) were often more exposed to the virus and represented more than half of the patients infected during the pandemic [3,4]. Moreover, few studies focused on determining the risk factors and risk markers associated with severity and mortality in this young population. Age is a risk marker for COVID-19 lethality, and young adults have a lower probability of death, indicating that they were not completely immune. A third of hospitalized patients in the United States were young adults [5], an age group whose infected had a rate of admission to intensive care units (ICUs) between 11 and 21 % and a mortality rate that could reach up to 5 % [6,7]. Some studies showed that mortality from COVID-19 increased due to the preexistence of comorbidities such as diabetes, obesity, and hypertension [8]. A mortality of up to 3.8 % has been observed in hospitalized young adults without any comorbidities [9].

The mortality associated with COVID-19 depends largely on the immune response triggered by the host against this betacoronavirus [10]. Recent studies suggest, for example, that the cytokine storm is caused mainly by macrophages and monocytes in inflammatory states [11]. In addition, the expansion of one or more of the monocytic sub-populations, classical (CD14^+^CD16^−^), intermediate (CD14^+^CD16^+^), or non-classical (CD14^dim^CD16^+^), has been associated with higher mortality in older adults [12,13].

Considering that little is known about the inflammatory and the phenotype profile of the various monocyte sub-populations (classical, intermediate, and nonclassical) in SARS-CoV-2 Positive young adults with a diagnosis of severe pneumonia, and its potential association with clinical outcomes, this study aims to identify whether monocyte dysregulation is a risk markers associated with mortality in this group of patients.

## 2 Materials and methods

### 2.1 Study design

Between January and June 2021, a prospective cohort study was carried out in a tertiary public university hospital with 428 general beds and 122 ICU beds (650 beds in total). This time frame corresponds to the third peak of the pandemic in Colombia, when the SARS-CoV-2 variant that circulated was B.1.621. During the enrolment period, COVID-19 vaccines were not available for population younger than 50 years old.

### 2.2 Patients

All consecutive adult subjects between the ages of 18 and 50 years, who were admitted with pneumonia diagnosis and confirmed to be infected with Acute Respiratory Syndrome Coronavirus 2 (SARS-CoV-2), were included in the study. Concurrently, a cohort of healthy young subjects was recruited for the purpose of comparing inflammatory markers, as reference levels in this population were previously unknown. The healthy controls exhibited no respiratory symptoms, tested negative for SARS-CoV-2 within 30 days before their enrollment, and provided informed consent for the collection of blood samples.

### 2.3 Confirmatory diagnosis

SARS-CoV-2 infection was confirmed by molecular detection techniques ID NOW COVID-19 *(*Abbott®, Chicago, USA) or VIASURE test SARS-CoV-2 Real Time PCR Detection Kit (San Mateo de Gállego, Spain) from nasopharyngeal swabs or bronchial secretions.

### 2.4 Routine biomarker measurement

Samples for routine biomarker assessment included D-dimer, C-reactive protein (CRP), LDH, ferritin, procalcitonin, lactic acid, fibrinogen, complete blood count, pH, and blood gas analysis. These samples were collected during the entire hospitalization period of the patients. The data presented in this report pertain to the analytes measured within the first 24 h of admission.

#### 2.4.1 Preservation of peripheral-blood mononuclear cells (PBMCs)

Whole-blood samples were acquired from both groups with heparinized tubes (Becton, Dickinson), and PBMCs were isolated by a Ficoll-Paque density-gradient centrifugation technique (Sigma-Aldrich, USA) for 20 min at 1,400*g*. After washing with PBS (pH 7.4), the isolated mononuclear cells were restored in fetal bovine serum (Sigma-Aldrich) with 10 % DMSO (Sigma-Aldrich) to be preserved in liquid nitrogen (−196 °C). Aliquots were stored in cryotubes (Sigma-Aldrich®) at 10 × 10^6^ cells/mL, which were quantified in a Neubauer chamber where cell viability ≥90 % was always verified by trypan blue (Thermo Fisher Scientific, USA). The cells were preserved in liquid nitrogen until use for Flow analysis.

#### 2.4.2 Serum analysis of inflammatory cytokines

Serum from patients and healthy controls were drawn into anticoagulant-free tubes (Becton, Dickinson), aliquoted, and stored at −80 °C. Interleukin (IL)-8, IL-6, and IL-10 were detected during the first 24 h of hospitalization using the Human Inflammatory Cytokines Kit (Becton, Dickinson and Co.). Standard curves, controls and samples were processed in a BD FACSCanto II flow cytometer (Becton, Dickinson and Co.) following the manufacturer's instructions. The results are presented in pg/mL, and the detection limits for each of the cytokines were 3.6 pg/mL (IL-8), 2.5 pg/mL (IL-6), and 3.3 pg/mL (IL-10). The raw files were processed with FCAP Array Software v3.0 (Becton, Dickinson and Co.) for analysis and quantification.

#### 2.4.3 Analysis of monocyte subpopulations

To perform the multiparametric analysis of monocytes by fluorescence-activated cell sorting (FACS), the following antibodies were used: CD14 (FITC), CD16 (PerCP-Cy), CD45 (V500), CD274 (PE-Cy7), and HLA-DR (PE), all purchased from Becton, Dickinson and Co. The PBMCs were incubated with stain buffer (BD) with the respective mix of antibodies for 30 min at 4 °C, protected from light. Cells were washed twice with stain buffer, centrifuged at 300*g* for 5 min and acquired by the FACSCanto II (Becton, Dickinson and Co.). Raw data were pooled on the FACSDiva platform (Becton, Dickinson and Co.), and fine population analyses were performed in FlowJo V10 software (Tree Star, USA). Compensation controls were performed by CompBeads (BD Biosciences, USA), and the acquisition controls were based on the fluorescence minus one (FMO) method. CD45^+^ monocytes were stratified as classical monocytes (CD14^+^CD16^−^), intermediate monocytes (CD14^+^CD16^+^), and non-classical monocytes (CD14^dim^CD16^+^). See the analysis plan and FMO controls in Supplementary Fig. 1.

### 2.5 Standard treatment

All patients with pneumonia received medical treatment according to the institutional protocols for this severe infection, following the scientific evidence known at the time of patient recruitment.

### 2.6 Outcomes to be studied

The outcome to be evaluated was the expression of the inflammatory biomarkers and the monocyte phenotype profile at discharge.

### 2.7 Ethics statement

All procedures followed the principles of the Declaration of Helsinki. This study was approved by the Research Ethics Committee of the Hospital Universitario del Valle (approval number 066–2020) and the Universidad del Valle (approval number: 016–020). Written informed consent was obtained from all participants or a family member.

### 2.8 Statistical analysis

The data were collected using the electronic data capture platform REDCap [14] hosted at the Universidad del Valle. Sociodemographic, clinical, and laboratory variables were gathered for all COVID-19 patients. In healthy volunteers, only sociodemographic and laboratory variables were gathered. Statistical analysis was performed in R version 4.2.2 (R Core Team, 2022) [15]. Qualitative variables are presented as absolute proportions and frequencies, and continuous variables are presented as mean ± standard deviation or median (interquartile range), depending on the distribution. Between-group comparisons were done by means of the chi-squared test, Student's *t*-test, U-Mann-Whiney, or Kruskal‒Wallis, according to the distribution of the data. p < 0.05 was considered statistically significant.

A bivariate analysis was performed to identify possible factors associated with mortality and severity. These were identified by drawing receiver operating characteristic (ROC) curves, and APACHE II score ≥12 was the cutoff to discriminate the most severe from the least severe.

Finally, the relative risk and its 95 % confidence intervals for mortality and severity were estimated among the cohort of subjects with severe pneumonia, according to the values of the biomarkers and clinical variables.

## 3 Results

### 3.1 Description of the population

Forty young adults (*n* = 40) diagnosed with severe pneumonia and confirmed acute SARS-CoV-2 infection were consecutively enrolled. Among them, approximately 70 % were male (28/40), with an average age of 43 ± 11.2 years (ranging from 36 to 50). Notably, none of the participants had received a SARS-CoV-2 vaccine. Among the patients with pneumonia, 22.5 % (9/40) had a medical history that included conditions such as obesity, chronic obstructive pulmonary disease (COPD), asthma, diabetes mellitus, or hypertension, with only one patient presenting more than one comorbidity.

### 3.2 Clinical results and routine biomarker behavior

The duration from symptom onset to admission was 10 ± 4.1 days, and the symptom progression was comparable between the survivors and those who passed away. Ninety percent (36/40) of the patients received corticosteroids from the initial hours of admission to the ICU, following the recommendations based on the preliminary findings of the RECOVERY trial [16] (see Table 1).

**Table 1:** Sociodemographic and clinical characteristics of patients with SARS-CoV-2 pneumonia.

	COVID-19 survivors n = 28		COVID-19 nonsurvivors n = 12		Total *n* = 40	p value ᵃ
Age (years)	47 [32.8–50]		4 [35.2–50]		47 [33–50]	0.9
Sex, M/F	17/11		11/1		28/12	0.1
Smoker	1 (3.6)		1 (8.3)		2 [5]	0.5
**Comorbidities**						
HT	2 (7.1)		1 (8.3)		3 (7.5)	1
Diabetes mellitus	1 (3.6)		0		1 (2.5)	1
COPD	1 (3.6)		0		1 (2.5)	1
Others	10 (35.7)		4 (33.3)		14 (35)	1
**At admission**						
APACHE II	6 [3–8.2]		12.5 [7.7–15.2]		7.0 [4–12]	<0.001
SOFA	3.39 ± 2		5.50	±2.4	4.02 ± 2.3	0.01
NEWS SCORE	7.03	±2.9	9.17	±3.3	7.67 ± 3.2	0.1
**Hospitalization**						
ICU stay	25 (89.3)		12 (100)		37 (92.5)	0.5
ICU stay (days)	7.5 [4.7–9.5]		9 [3–16]		8 [4–11]	<0.001
**At discharge**						
Mortality rate					30 %	

Data are shown as mean ± standard deviation, median [interquartile range], or percentage. ᵃ Student's *t*-test, Mann‒Whitney's *U* test, chi-squared test, or Fisher's, test as appropriate.

Upon admission, 30 % of the participants had an APACHE II score of ≥12 points (Table 1). The overall mortality rate was 30 % (12/40) (Table 1). Sixty percent of the patients (24/40) required invasive mechanical ventilatory support. Furthermore, the duration of ICU stay was longer among the deceased patients in comparison to the survivors (9 days vs. 7.5 days, p < 0.001).

Deceased patients exhibited significantly lower peripheral lymphocyte counts and higher levels of C-reactive protein (CRP), lactate dehydrogenase (LDH), and procalcitonin in comparison to the surviving patients (see Table 2). No notable differences were observed in D-dimer, ferritin, sex, or age (Table 2).

**Table 2:** Biomarkers upon hospital admission, according to discharge status.

	COVID-19 survivors n = 28	COVID-19 non survivors n = 12	Total *n* = 40	p value ᵃ
Clinical laboratories				
Leukocytes ( × 10^3^/μL)	11.4 [6.6–14.2]	10.5 [7.7–18.9]	11.1 [6.7–15.1]	0.57
Lymphocytes ( × 10^3^/μL)	1.2 [0.9–1.7]	0.7 [0.5–0.1]	1.1 [0.7–1.6]	**0.03**
Monocytes (%)	5.2 [3.8–7.5]	3.6 [2.1–4.7]	4.7 [3.1–6.2]	**0.01**
Lymphopenia	13 (59.1)	9 (40.9)	22 (100)	0.19
CRP (ml/L)	87.3 [51.1–213]	241.4 [128–356.7]	159.1 [60.4–233.7]	**0.008**
LDH (U/L)	364 [318.8–533.2]	634.5 [405.2–1132.8]	429.5 [329.2–640.5]	**0.03**
AST transaminases (U/L)	60 [38–68]	58.5 [46.2–106.2]	60 [40–85]	0.37
Ferritins (ng/mL)	651 [252–1240]	690.5 [22.3–1320]	651 [252–1240]	0.86
Procalcitonin (ng/mL)	0.3 [0.1–0.7]	0.8 [0.3–3.5]	0.3 [0.2–1.1]	**0.03**
D-dimers (mg/L)	0.9 [0.5–1.8]	1.4 [0.9–2.8]	1.2 [0.7–2.2]	0.08
Fibrinogen (ml/dL)	417.4 ± 133.2	367.8 ± 109.5	405.7 ± 126.6	0.48
Lactic Acid (ml/dL)	1.9 [1.4–2.4]	1.9 [1.5–2.5]	1.9 [1.4–2.5]	0.77
pH *	7.4 [7.4–7.4]	7.3 [7.3–7.5]	7.4 [7.3–7.4]	0.26

Data are shown as mean ± standard deviation, median [interquartile range], or percentage.

Student's *t*-test or Mann‒Whitney's, *U* test as appropriate.

### 3.3 Levels of the inflammatory cytokines IL-6, IL-8, and IL-10

Deceased patients had higher IL-6, IL-8, and IL-10 cytokines levels than survivors (Table-3). IL-6 was nine times increased in the deceased compared to survivors (159.73 [89.6–639] pg/mL vs. 17.90 [7–38.5] pg/mL). Similarly, IL-8 and IL-10 were elevated (3-fold increase) in the deceased than survivors (Table-3). These differences were even more marked when the concentrations of both survivors and deceased were compared with respect to healthy controls (Table 3). Furthermore, the cytokines evaluated were higher in the critically ill patients (APACHE II ≥ 12) than in the less severe individuals (APACHE II < 12).

**Table 3:** Expression of cytokines and monocyte status in COVID-19 patients at discharge or death compared with the cohort of healthy young people.

	COVID-19 survivors n = 28	COVID-19 non survivors n = 12	Healthy controls n = 20	p value ᵃ
**Cytokines (ng/dL)**				
Human IL-10	4.1 [1.9–5.8]	13.4 [7.4–22.8]	1 [0.8–1.3]	<0.001
Human IL-6	17.9 [6.9–38.5]	159.7 [89.6–639.1]	2.2 [1.6–2.9]	<0.001
Human IL-8	32.7 [21.3–62.6]	153.2 [91–181.7]	13.9 [11.6–18.1]	<0.001
**HLA-DR expression on monocytes**				
classical monocytes	39.8 ± 21.3	22.2 ± 16.1	34.7 ± 21.3	**0.02**
intermediate monocytes	17.6 [8.5–29.7]	8.1 [5–26.9]	17.2 [7.3–28.7]	0.24
nonclassical monocytes	6 [4.2–8.4]	4.8 [2.4–6.8]	5.9 [3.8–9.7]	0.24
Global monocytes CD14^+^	39.2 ± 22.1	26.6 ± 17.2	35.5 ± 21.4	0.11
**PD-L1 expression on monocytes**				
classical monocytes	77.8 [72–83]	72.7 [62.6–79]	76 [70.6–82.8]	0.12
intermediate monocyte	75.3 ± 14.4	74.7 ± 10.2	75.2 ± 13.2	0.78
nonclassical monocytes	37.9 ± 12.7)	37.2 ± 9.9	37.7 ± 11.8	0.63
Global monocytes CD14^+^	79.6 [72.2–85.5]	72.3 [62.9–81.7]	77.2 [70–84.9]	0.14

Data are shown as mean ± standard deviation or median [interquartile range].

ᵃ Student's *t*-test or Mann‒Whitney-Whitney, *U* test as appropriate.

#### 3.3.1 Evaluation of HLA-DR and PD-L1 expression in monocyte subpopulations of COVID-19 patients compared to healthy controls

**Fig. 1:**
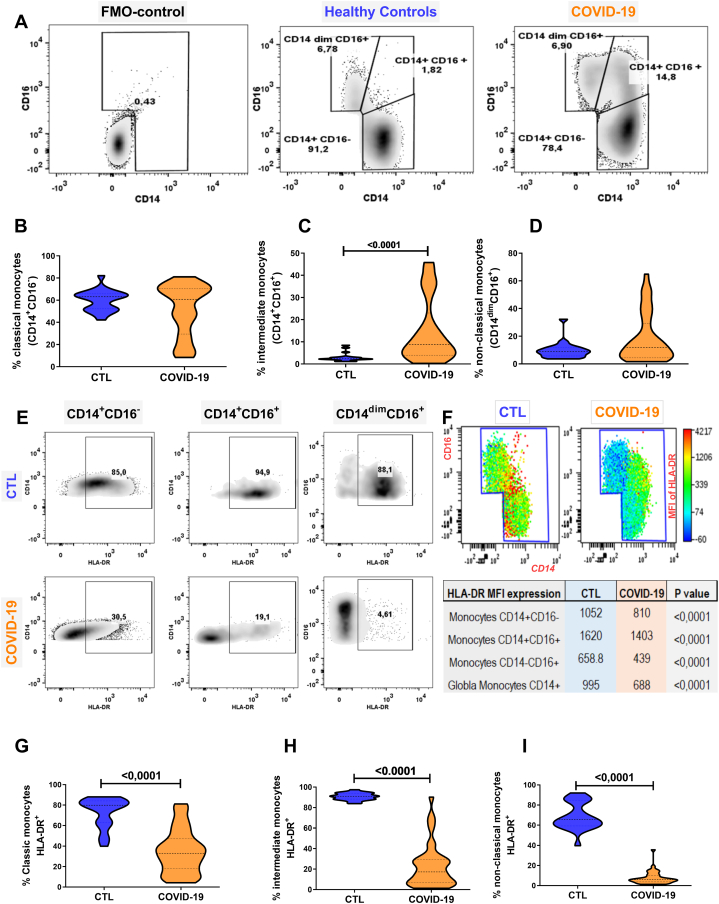
Quantification of classical, intermediate and nonclassical monocytes and their HLA-DR expression in healthy controls vs. young patients with severe COVID-19. (A) Fluorescence minus one control (FMO) and representative FACS plots of monocyte subpopulations (Classical, Intermediate, and nonclassical) in healthy controls and young patients with COVID-19. (B–D) violin plots illustrating the comparative distribution of classical (CD14^+^ CD16^−^), Intermediate (CD14^+^ CD16^+^), and non-classical (CD14^dim^ CD16^+^) monocytes between healthy controls and young patients with COVID-19. (E) Assessment of HLA-DR expression on monocyte subtypes in healthy controls and young patients, followed by (F) presentation of mean fluorescence intensity (MFI) distribution of HLA-DR on monocyte subtypes. (G–I) violin plots highlighting comparative HLA-DR expression in classical (CD14^+^ CD16^−^), intermediate (CD14^+^ CD16^+^), and non-classical (CD14^dim^ CD16^+^) monocytes between healthy controls and young patients with severe COVID-19. ^#^ Student's *t*-test or Mann‒Whitney-Whitney, *U* test as appropriate.

Absolute monocyte count was within the normal reference range for the young population in all the study groups (Table 2). However, flow cytometry analysis revealed than patients with COVID-19 severe pneumonia presented a significant expansion of the intermediate CD14^+^CD16^+^ monocytes subsets when compared to healthy controls (Fig. 1. A and C). On the other hand, the classical CD14^+^CD16^−^ monocytes and non-classical CD14^dim^CD16^+^ monocytes showed no differences when compared with healthy controls (Fig. 1. A, B and D).

**Fig. 2:**
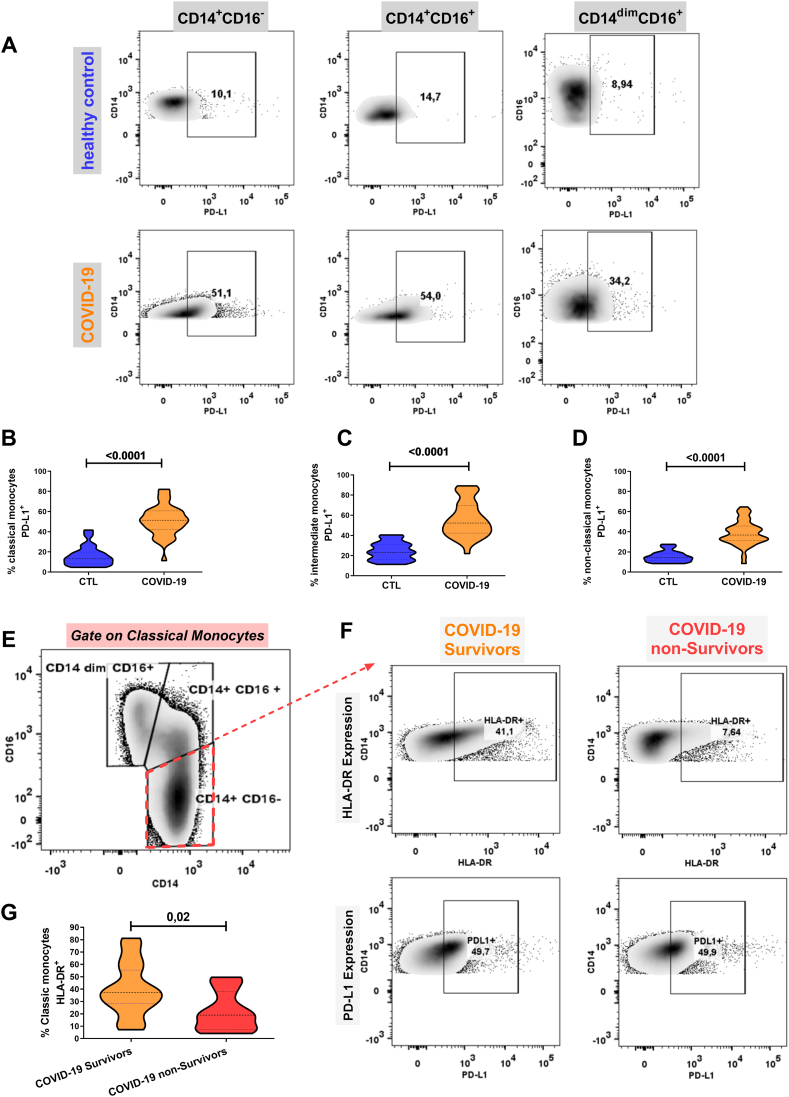
Expression of PD-L1 in monocyte subsets and quantification of HLA-DR in classical monocytes (CD14^+^CD16^−^) of surviving vs. nonsurviving patients with severe COVID -19 pneumonia. (A) Flow cytometry plots illustrating PD-L1 expression on monocyte subsets: Classical (CD14^+^CD16^−^), intermediate (CD14^+^CD16^+^), and non-classical (CD14^dim^CD16^+^) monocytes in control subjects and young patients with COVID-19.(B–D) Comparative expression of PD-L1 depicted as violin plots among classical, intermediate, and non-classical monocyte subsets in control subjects and young patients with COVID-19. (E) Flow cytometry plot demonstrating gate selection on classical monocytes from COVID-19 patients, followed by (F) subplots displaying expression profiles of HLA-DR and PD-L1 on classical monocytes in surviving versus non-surviving patients with severe COVID-19. (G) Violin plot displaying relative expression levels of HLA-DR in a comparison between surviving and non-Surviving Patients. ^#^ Student's *t*-test or Mann‒Whitney-Whitney, *U* test as appropriate.

COVID-19 patients had a significantly lower frequency of HLA-DR expression in all monocyte subpopulations than healthy controls (Fig. 1. E, G-I). These results were supported by the decrease in the mean fluorescence intensity (MFI) on these subpopulations (Fig. 1. F). Additionally, marked expression of PD-L1 (a transmembrane protein associated with anergy) was observed in the classical, intermediate, and non-classical monocyte subpopulations of all patients with COVID-19 (Fig. 2 A-D).

#### 3.3.2 Evaluation of HLA-DR and PD-L1 expression in monocyte subpopulations of young adults who survived and did not survive severe cases of COVID-19

No differences were observed in the expansion of monocyte subpopulations (classical, intermediate, and nonclassical) between survivors and non-survivors of severe COVID-19 (Supplementary Fig.2, A-C). However, a significant reduction in the frequency of HLA-DR expression was found in classical monocytes of deceased patients compared with survivors (22.2 % vs. 39.8 %, P = 0.02, respectively, see Table 3) (Fig. 2, E-G), as well as in those with more severe forms of the disease (Supplementary Table 1).

**Fig. 3:**
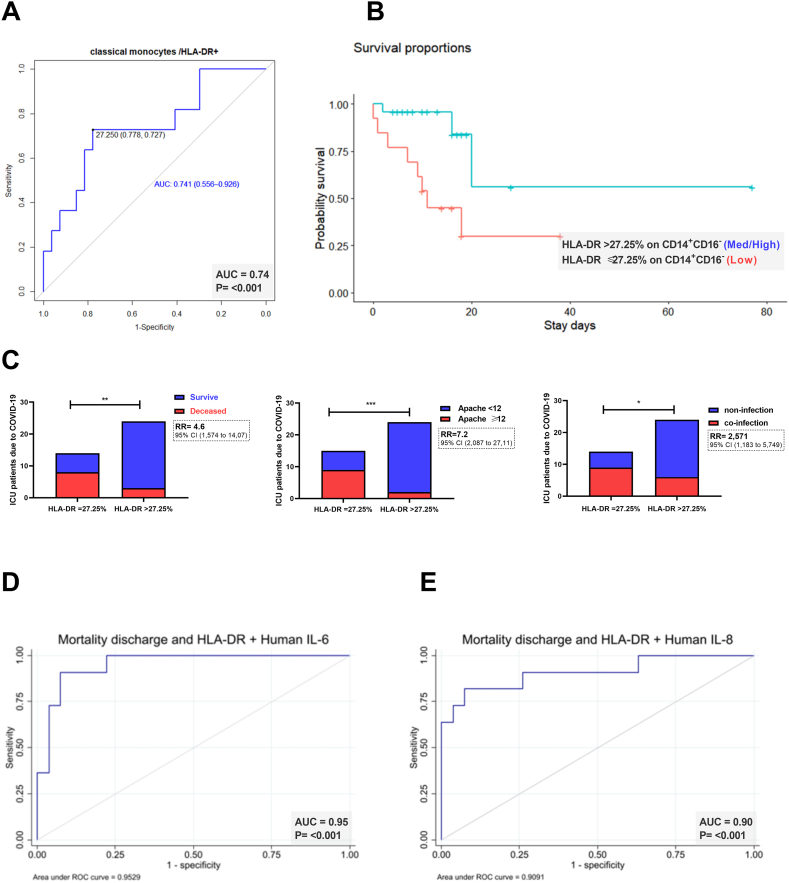
A significant reduction in the frequency of expression of HLA-DR in classic monocytes from patients with COVID-19 is associated with mortality, severity, and bacterial infection. (A) Receiver operating characteristic analysis (ROC) showed the predictive ability of HLA-DR expression in classic monocytes from COVID -19 patients, yielding an area under the curve (AUC) of 0.74 for mortality prediction in young individuals with severe COVID -19. (B) Kaplan–Meier survival curves showed survival probabilities as a function of HLA-DR expression levels 27.3 % or 27.3 % in classic monocytes from severe COVID -19 (C) Comparative bar graphs showed the incidence of mortality, disease severity (APACHE 12), and coinfection in the subgroup of severe COVID -19 patients depending on whether HLA-DR expression levels in classic monocytes were 27.3 % or 27.3 %. (D–E) Analysis by ROC shows the predictive efficacy of pooling expression of classic monocytes HLA-DR with serum IL -6 or IL -8 in young patients with severe COVID-19. (*P < 0.05, **P < 0.01, ***P < 0.001).

We also observed significant differences in the frequency of HLA-DR expression in classical monocytes between our population of young patients without comorbidities (n = 31) who survived and those who did not survived after SARS-CoV-2 infection (HLA-DR expression: 37.6 % vs. 19.40 %, P = 0.02, respectively). Similarly, the lower HLA-DR expression was associated with the higher probability of death (Fig. 3. A, B); thus, HLA-DR ≤ 27.3 % in classical monocytes generated a relative risk for mortality of 4.6 (95 % CI, 1.6–14.1), a risk for severity of 7.2 (95 % CI, 2.1–27.1), and a risk for ICU coinfection of 2.6 (95 % CI, 1.2–5.7) (Fig. 3. C).

Although PD-L1 was elevated in the monocyte subpopulations of COVID-19 patients, we did not find differences in the expression of this protein between non-survivors and survivors patients (Table 3 and Supplementary Fig.2, D-F). Additionally, no significant differences were found in the expression of HLA-DR on intermediate or non-classical monocytes when comparing the groups of survivors and non-survivors (Table 3).

Finally, in deceased patients, we found an inflammatory profile with of elevated expression of interleukins IL-6 and IL-8, together with the low expression of HLA-DR in classical monocytes. This profile could be an important biomarker for predicting death in SARS-CoV-2+ young adults who were hospitalized in the ICU (Fig. 3. D, E).

## 4 Discussion

Unvaccinated young adults (up to 50 years old) who had confirmed severe pneumonia due to COVID-19, included in our study presented 30 % (12/40) mortality rate. Those who died of the disease had higher IL-6, IL-8, and IL-10 together with suboptimal expression of HLA-DR in their classical monocytes. These findings indicate severe monocyte immunosuppression that was accompanied by lymphopenia, which largely explains the outcomes of these young patients.

Monocytes circulating in the blood can provide valuable information about the inflammatory status of the host [17]. Recent evidence indicates that both monocytes and macrophages play an important role in the resistance, pathogenesis, and resolution of COVID-19 [18]. These cells recognize viral RNA through recognition receptors such as TLR3, TLR7, and TLR8 and respond to infection by secreting inflammatory cytokines [19]. When there is an excess of inflammatory mediators, a cytokine storm occurs, which is largely responsible for the pneumonia, coagulopathy, and septic shock observed in critically ill patients [20].

Although no monocytopenia or differences in the absolute values of monocytes were detected between our surviving vs. deceased patients diagnosed with COVID-19, an anomaly in the normal distribution of monocyte populations was observed by flow cytometry. Specifically, intermediate monocytes (CD14^+^CD16^+^) of young adults with a severe diagnosis of COVID-19 were more abundant than those found in healthy controls. Solid evidence shows that the expansion of intermediate monocytes is associated with serious viral infections, such as dengue with warning signs and West Nile fever [21]. In COVID-19, Zhou et al. showed that patients admitted to the ICU had a greater monocyte CD14^+^CD16^+^ proportions than those not admitted to the ICU. Using intracellular marking technique, they confirmed that these cells produced IL-6 and granulocyte–macrophage colony-stimulating factor, which suggests that these cells could be related to the pulmonary inflammatory pathology and the clinical manifestations observed in more severe patients [22].

In our study, patients who had high serum levels of proinflammatory cytokines were also the most severe patients and also those who presented higher mortality rate. This inflammatory response was characterized by elevated serum levels of cytokines, including IL-6, IL-8, and IL-10, as well as acute-phase reactants such as PCR and LDH. Notably, when analyzed collectively, these markers showed a significant increase in patients who did not survive. Furthermore, our observations revealed that patients necessitating invasive mechanical ventilation exhibited higher levels of inflammatory cytokines (See Supplementary Table 2) These findings could imply an exaggerated immune response to SARS-CoV-2 in younger patients, possibly driven by the expansion of CD14+CD16^+^ monocytes.

However, it should be noted that there is currently no consensus on the pattern of monocyte populations that are enriched or not in COVID-19 patients. In the literature, contradictory findings can be found with other investigations, where some groups have shown, for example, a reduction of the intermediate subpopulation [23] or expansion of classical monocytes in severe SARS-CoV-2 infections [11]. These differences may be due substantially to variations in terms of definition of severity, observation time, and FACS protocols but, more importantly, to their study of the subpopulation of young patients with no or minimal comorbidities. However, a common and generalizing element that we can highlight in some investigations is the possible monocyte dysfunction found in severe patients, which has been marked by the reduction of major histocompatibility complex (MHC)-II [24], as well as by the low expression of other stimulatory molecules such as CD40 and CD86 [25].

Alterations in MHC-II could indicate a lower capacity for antigen presentation and T-cell activation, which in turn could be related to more marked immunosuppression, mainly in deceased patients. In fact, and reinforcing the above, the deceased patients presented lower lymphocyte values and higher serum concentrations of IL-10 than those patients who survived, who also had a higher risk of bacterial co-infections when the classical monocytes presented suboptimal values of HLA-DR (≤27.2 %). The reduction of HLA-DR in CD14^+^CD16^−^ monocytes is reliably associated with an increased risk of bacterial confections in critically ill patients. Monneret et al. demonstrated that a proportion of CD14^+^ monocytes presenting HLA-DR ≤30 % was an independent predictor of mortality in patients with septic shock [26,27].

Currently, the underlying causes of monocyte anergy in COVID-19 are not fully understood. Overproduction of cytokines in the context of COVID-19 can deregulate monocyte function. IL-10 can inhibit the activity of the JAK2 enzyme, which is essential for the activation of the JAK-STAT signaling pathway and therefore reduces the expression of HLA-DR [28,29]. Furthermore, IL-6 may affect the ability of human dendritic cells to activate specific CD4^+^ T cells via a reduction in MHC-II [30]. High levels of IL-6 could be a possible cause of decreased expression of monocyte HLA-DR in patients with severe COVID-19. It is possible that the hyperinflammatory response associated with SARS-CoV-2 generates immunological dysfunction at the level of monocytes and lymphocytes, similar to what has been observed in sepsis. Sepsis is a syndrome in which innate immunity and inhibitory cytokine production are altered, so it can contribute to the dysfunction of multiple organs and thereby increase mortality in the ICU [31], though other studies have indicated that mortality in these patients was associated with the presence of comorbidities, specifically obesity and diabetes mellitus [9]. The mortality of 30 % in this subgroup of patients was associated with the high lethality of the variant that circulated in the third peak of the pandemic and the high exposure to infection in the young population given the coincidence with the social moment that existed in Colombia.

Our research also revealed that young adult patients admitted to the intensive care unit due to SARS-CoV-2 exhibited a pronounced expression of PD-L1 on their monocytes. Although we did not find a direct association between the high expression of PD-L1 and mortality in our study, this heightened immunological marker could be related to the severity and complexity of the patients included in the study. In fact, in the context of COVID-19, several studies have demonstrated that severe or critical patients show an increase in PD-L1 in dendritic cells and monocytes, and it has even been detected in a soluble form in critical patients [33]. While the exact role of the PD-1/PD-L1 axis in the pathogenesis of COVID-19 is still not completely clear, it is well-known that PD-L1 acts as a potent negative regulator of T-cell activation [33], which could also explain the lymphopenia we observed in our results.

The following limitations of this study should be acknowledged. The cohort was taken from a single health care center, which may hinder the generalization of the results to other populations. The cohort of healthy volunteers consisted of asymptomatic subjects, without comorbidities and with negative tests for SARS-CoV-2 in times of a pandemic, which could introduce a bias in the sense that although asymptomatic and with negative tests at the time of the blood sample, they could have had a minimal inflammatory response. In any case, the immunological profiles of the healthy volunteers were always lower than those of the sick volunteers. On the other hand, a significant strength of the study was its immunological examination of the outcome of an unvaccinated young adult population, which had severe forms of COVID-19 and a low rate of comorbidities. This provided valuable information on the immune responses in a specific group of patients, which is relevant to improving our understanding of the disease in this subset of people.

In young patients with severe forms of COVID-19, an inflammatory phenotype combined with monocyte immunosuppression was observed, a phenomenon that could largely explain the severity of respiratory symptoms. The most notable was the strong reduction in HLA-DR on classical monocytes, which was correlated with higher mortality and a longer hospital stay in the ICU. To date, even after the pandemic has been overcome, this study is the first to evaluate monocyte status in young adults not vaccinated for COVID-19 who had severe pneumonia and few or no comorbidities. In light of the current results, we still do not know the long-term effects of hyperinflammation in young survivors of severe forms of COVID-19.

Finally, this panorama generates several challenges in the face of new variants of COVID-19 or potential viruses that could generate pandemics and systemic inflammation in the infected. First, it is necessary to carry out in-depth studies to understand the molecular and immunological mechanisms behind this monocyte dysregulation and thus generate therapeutic proposals in which the functional capacity of monocytes and macrophages can be repowered again, not only in COVID-19 but probably in other sepsis with viral characteristics. Second, there is a growing need to evaluate cytokines and monocyte populations in clinical practice. This study was done with the intention of gaining a better diagnostic impression of the inflammatory and functional status of patients in the ICU. Although the benefits of measuring monocyte HLA-DR in critically ill patients have been demonstrated, this has not yet translated into global clinical practice. Nevertheless, in light of these findings and the evidence provided by other researchers, we believe it is relevant to assess the behavior of monocyte HLA-DR in critically ill patients associated with viral or bacterial sepsis. This assessment aims to monitor the degree of innate immunosuppression. Some clinical approaches may be linked to the use of agonists that could assist in or restore monocyte function. Furthermore, monocyte subpopulations into clinical practice is crucial for obtaining a more comprehensive diagnostic assessment of the inflammatory and functional status of critically ill patients. For instance, a recent case report showed that interferon-γ treatment led to a significant improvement in CD14^+^ monocyte HLA-DR levels, providing an improvement in the clinical status of a patient with severe COVID-19 and a diagnosis of severe sepsis with multiple multiresistant bacterial confections [32].

## 5 Conclusion

Our study reveals that young adult patients with severe COVID-19 pneumonia exhibit an inflammatory profile alongside monocyte immunosuppression, characterized by a significant reduction in HLA-DR expression within classical monocytes. This reduction in HLA-DR is positively correlated with clinical severity, bacterial coinfection, and mortality. These findings lend substantial support to the hypothesis that monocyte immunosuppression plays a pivotal role in the pathogenesis of SARS-CoV-2. Further investigations are imperative to elucidate the underlying molecular and immunological mechanisms associated with monocyte dysregulation, thus contributing to the identification of potential therapeutic interventions aimed at revitalizing monocyte and macrophage function in the context of viral sepsis.

Furthermore, integrating the routine evaluation of cytokines and monocyte subpopulations into clinical practice is crucial for obtaining a more comprehensive diagnostic assessment of the inflammatory and functional status of critically ill patients. It is noteworthy that our study represents the first to establish a direct association between low HLA-DR expression in classical monocytes and increased mortality risk in young patients with severe COVID-19.

## Funding

This study was supported by 10.13039/501100007329Universidad del Valle in 2020 to contribute to the knowledge and mitigation of the COVID-19 impact (grant number 1896) and UnidadCentral del Valle del Cauca (grant number 14-2020). This was funded in part by the Division of Intramural Research (DIR), 10.13039/100000060NIAID, NIH.

## Data sharing statement

A version of the database (anonymized) and laboratory protocols are available on request. Inquiries should be directed to jshenao@uceva.edu.co or lorena.matta@correounivalle.edu.co.

## CRediT authorship contribution statement

**Juan Sebastián Henao-Agudelo:** Writing – review & editing, Writing – original draft, Validation, Project administration, Methodology, Investigation, Funding acquisition, Formal analysis, Data curation, Conceptualization. **Sebastian Ayala:** Writing – original draft, Supervision, Resources, Investigation. **Marisol Badiel:** Writing – review & editing, Writing – original draft, Validation, Methodology, Formal analysis, Data curation, Conceptualization. **Andrés F. Zea-Vera:** Writing – review & editing, Writing – original draft, Formal analysis, Data curation, Conceptualization. **Lorena Matta Cortes:** Writing – review & editing, Writing – original draft, Supervision, Project administration, Methodology, Funding acquisition, Formal analysis, Conceptualization.

## Declaration of competing interest

The authors declare that they have no known competing financial interests or personal relationships that could have appeared to influence the work reported in this paper.
