# Supplementary material for: Classical monocytes-low expressing HLA-DR is associated with higher mortality rate in SARS-CoV-2+ young patients with severe pneumonia
Source: Heliyon. 2024 Jan 9;10(2):e24099. doi: 10.1016/j.heliyon.2024.e24099 (PMC10803910; doi:10.1016/j.heliyon.2024.e24099)
Supplement: Multimedia component 1 [file mmc1.docx]

**Classical monocytes-low expressing HLA-DR is associated with higher mortality rate in SARS-CoV-2+ young patients with severe pneumonia.**

Juan Sebastián Henao-Agudelo ^1^, Sebastian Ayala ^2.3^, Marisol Badiel ^2.3^, Andrés F. Zea-Vera ^4^, Lorena Matta Cortes ^2.3^.

1. Faculty of Health Sciences, Central Unit of Valle del Cauca, Tuluá, Colombia.

2. Department of Internal Medicine, Universidad del Valle, Cali, Colombia.

3. Evaristo García Valley University Hospital, Cali, Colombia

4. School of Basic Sciences, Universidad del Valle, Cali, Colombia.


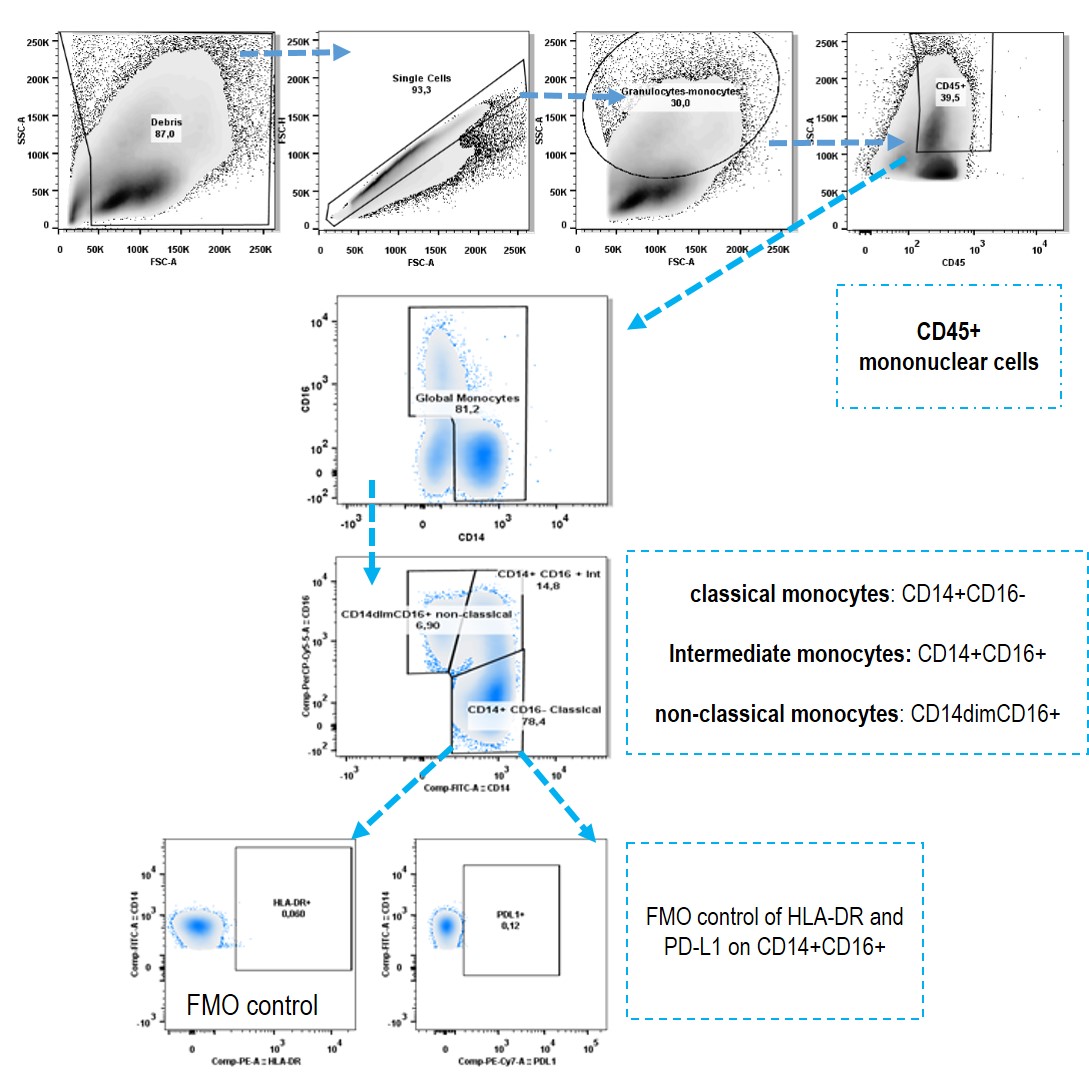


**Supplementary Figure 1. Flow cytometry gating strategy for the analysis of monocyte subpopulations in healthy controls and covid-19 patients with severe pneumonia.** Monocytes were gated with CD45 and subsets are dissected with a CD14/CD16 plot. HLA-DR and PD-L1 expressions were assessed on classical, intermediate, and non-classical monocytes. Flurescen minos one (FMO) control strategy was applied to gates requiring fluorochromes.


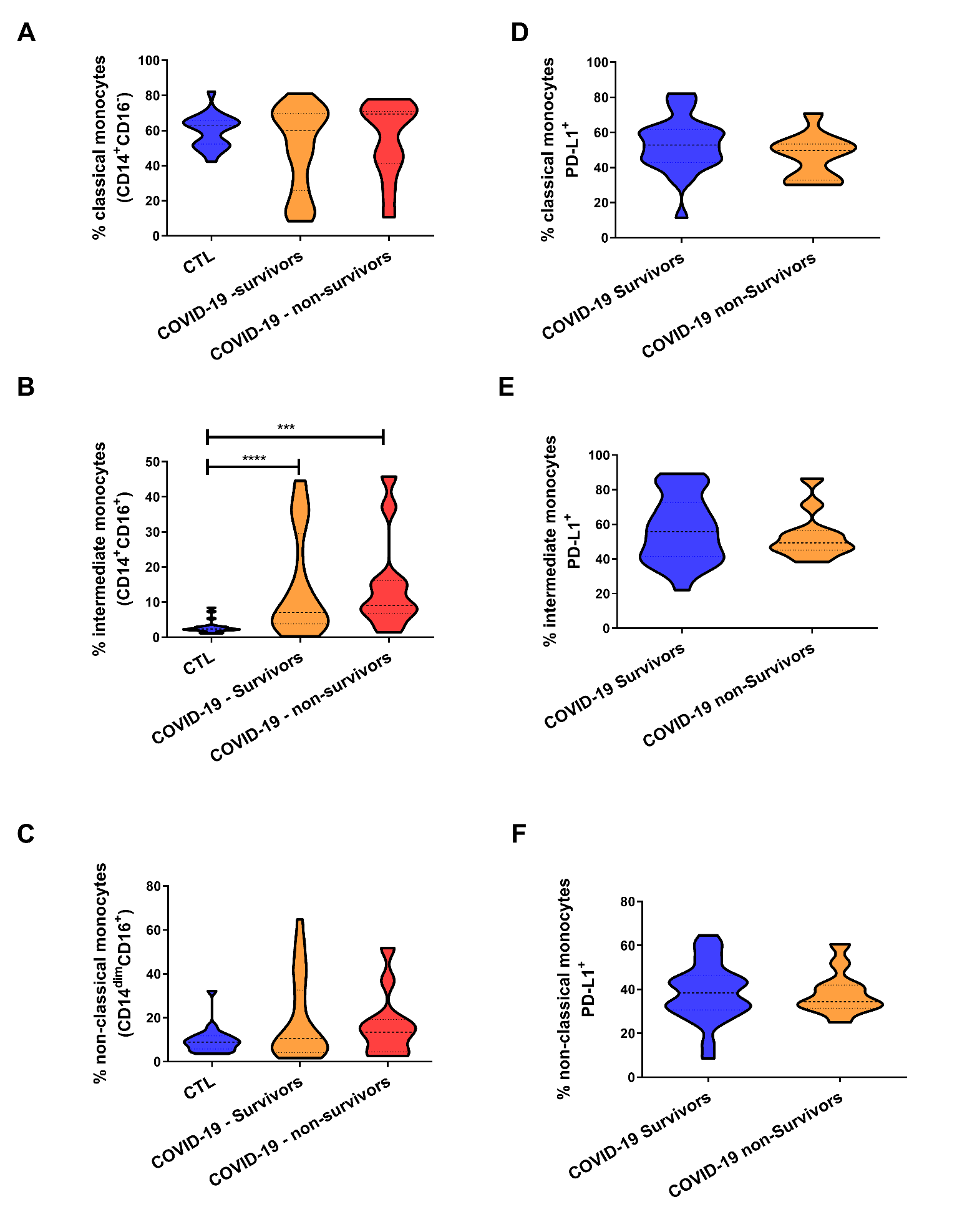


**Supplementary Figure 2. Quantification of classical, intermediate and nonclassical monocytes in COVID-19 patients and PD-L1 expression between survivors and nonsurvivors. (A-C)** Violin plots depict the comparative distribution of classical (CD14^+^ CD16^-^), intermediate (CD14+ CD16+), and non-classical (CD14^dim^ CD16^+^) monocytes among healthy controls, COVID-19 survivors, and nonsurvivors. **(D-F)** PD-L1 expression on monocyte subtypes is illustrated through violin plots, highlighting distinctions between survivors and nonsurvivors. (*P < 0.05, **P < 0.01, ***P < 0.001).

**Supplementary Table 1.**  Monocyte Status and Risk Scores Quantification in COVID-19 Patients with Severe Pneumonia, Stratified by APACHE II Scores (<12 or ≥12).

| **Variables** | **Apache II Score <12** | **Apache II Score ≥12** | **Total** | **p-value** |
| --- | --- | --- | --- | --- |
| N | 28 (70) | 12 (30) | 40 (100) |  |
| **Sex-no. (%)** |  |  |  |  |
| Man | 20 (71.4) | 8 (28.6) | 28 (70.00) | 1 |
| Woman | 8 (66.7) | 4 (33.3) | 12 (30.00) |  |
| **APACHE** | 5.50 (3.00-7.25) | 14.00 (12.75-16.25) | 7.0 (4.0-12.0) | **<0.001** |
| **SOFA** | 3.5 (1.64) | 5.25 (3.19) | 4.02 (2.75-5.00) | 0.11 |
| **New score** |  |  |  |  |
| **0 a 4 (Low)** | 6 (100) | 0 | 6 (15.0) | **0.02** |
| **5 a 6 (Medium)** | 7 (87.5) | 1 (12.5) | 8 (20.0) |  |
| **>7 (High)** | 15 (57.7) | 11 (42.3) | 26 (65.0) |  |
| **HLA-DR expression on Monocytes** |  |  |  |  |
| **classical monocytes /HLA-DR+** | 41.03 (21.23) | 19.20 (11.70) | 34.71 (21.31) | **<0.001** |
| **intermediate monocytes/HLA-DR+** | 15.40 (7.86-28.55) | 19.00 (3.29-29.30) | 17.25 (7.29-28.67) | 0.63 |
| **non-classical monocytes/HLA-DR+** | 5.98 (3.82-9.52) | 5.22 (3.41-9.52) | 5.96 (3.81-9.70) | 0.85 |
| **PD-L1 expression on Monocytes** |  |  |  |  |
| **classical monocytes/PDL1+** | 76.20 (70.65-83.00) | 75.90 (68.35-81.45) | 76.05 (70.62-82.78) | 0.56 |
| **intermediate monocyte/PDL1+** | 73.99 (13.97) | 78.03 (11.06) | 75.16 (13.18) | 0.35 |
| **non-classical monocytes/ PDL1+** | 36.86 (11.92) | 39.85 (11.92) | 37.73 (11.84) | 0.49 |

**Supplementary Table 2: Inflammatory Cytokine Expression According to the Need for Invasive Mechanical Ventilation.**

|  |  |  |  |  |
| --- | --- | --- | --- | --- |
| ***Necessity for Mechanical Ventilation*** | | | | |
| ***Cytokines*** | **No ventilation** | **Ventilation** | **healthy controls** | **p-value** |
| **Human IL-10** | 3.75 (2.07-7.78) | 6.26 (4.19-13.21) | 1.04 (0.76-1.32) | **<0.001** |
| **Human IL-6,** | 23.02 (5.67-55.96) | 49.51 (13.18-134.08) | 2.23 (1.60-2.90) | **<0.001** |
| **Human IL-8** | 29.77 (20.27-71.50) | 64.40 (31.93-107.56) | 13.92 (11.63-18.06) | **<0.001** |
